# *MPL*-mutated essential thrombocythemia: a morphologic reappraisal

**DOI:** 10.1038/s41408-018-0159-3

**Published:** 2018-11-20

**Authors:** Natasha Szuber, Curtis A. Hanson, Terra L. Lasho, Christy Finke, Rhett P. Ketterling, Animesh Pardanani, Naseema Gangat, Ayalew Tefferi

**Affiliations:** 1 Division of Hematology, Departments of Internal and Laboratory Medicine, Mayo Clinic, Rochester, MN USA; 2 Division of Hematopathology, Departments of Internal and Laboratory Medicine, Mayo Clinic, Rochester, MN USA; 3 Division of Laboratory Genetics and Genomics, Departments of Internal and Laboratory Medicine, Mayo Clinic, Rochester, MN USA

Our insight into the molecular basis of myeloproliferative neoplasms (MPN) took a landmark stride in 2005 with the identification of *JAK2* mutations in nearly all polycythemia vera and the majority of essential thrombocythemia (ET) and primary myelofibrosis (PMF) cases^1–3^. The subsequent identification of novel somatic activating mutations in myeloproliferative leukemia virus oncogene (*MPL*) in 2006 provided an additional mechanistic explanation for JAK–STAT activation in MPN^4^. Differential distribution of *MPL* mutations according to MPN subtype has since been discerned: *MPL*-mutated ET is uncommon, with a variable but typically cited incidence of less than 5% (5–9), while that of *MPL*-mutated PMF is at least twice as frequent^5^. Phenotypically, older age and lower hemoglobin levels have been remarked in *MPL*-mutated vs unmutated ET cohorts^6,7^. Further, *MPL*-mutated ET cohorts have higher reported rates of fibrotic progression than their *MPL* wild-type counterparts: in the order of 33.3% (vs 7.5% in *MPL*-unmutated) in one series^8,9^. Together, these observations suggest the possibility that some instances of *MPL*-mutated ET might actually represent prefibrotic PMF. This distinction is not insignificant as prognosis and management may vary correspondingly^10^. The current study was designed as a histopathological reappraisal and retrospective assessment of phenotypic and prognostic correlates in consecutive cases designated as *MPL*-mutated ET.

After board approval, patients were recruited from the institutional databases of the Mayo Clinic, Rochester, MN, USA. Study inclusion criteria required the availability of bone marrow biopsy slides obtained at diagnosis or within 1 year of diagnosis for central review by one of the authors (C.A.H.), an experienced hematopathologist. Central pathology review included assessment of bone marrow cellularity and extent of trilineage proliferation, megakaryocyte morphology, and grading of reticulin fibrosis. The review process was completely blinded to clinical, laboratory, and outcomes data. All diagnoses were in accordance with the 2016 World Health Organization (WHO) criteria^11^; the diagnosis of prefibrotic PMF specifically was based on strict morphological criteria as the stipulation for blinded review precluded access to clinical data. Screening for driver mutation status was performed using conventional methods. Data abstracted corresponded to the time of diagnosis, or within 1 year, and included *MPL* mutation type, peripheral blood smear parameters, karyotype, and presence of additional non-driver mutations based on availability of next-generation sequencing-derived information. Corresponding data were collected from *MPL*-mutated patients with PMF for purpose of comparison. Risk stratification was consistent with conventional prognostic models: international prognostic score for ET (IPSET)^12^ and dynamic international prognostic scoring system for PMF (DIPSS-plus)^13^. Overall survival (OS) was defined by the time from date of referral to date of death (uncensored) or last contact (censored); myelofibrosis-free survival (MFFS) considered myelofibrotic transformation as the uncensored variable. Differences in the distribution of continuous variables between categories were compared using the Mann–Whitney or Kruskal–Wallis test. Categorical variables were compared using the *χ*^2^ test. Survival and time-to-event curves were prepared using the Kaplan–Meier method and compared by the log-rank test. *P*-values <0.05 were considered significant. The JMP® Pro 13.0.0 software package was used for all analyses (SAS Institute, Cary, NC, USA).

A total of 665 patients with ET were annotated for their driver mutational status; 18 (2.7%) were reported out as being *MPL*-mutated; by comparison, among 867 patients with PMF, 47 (5.4%) were signed out as *MPL*-mutated. Among the 18 cases with *MPL*-mutated ET, bone marrow sides were available for central pathology review in 14 patients; all but 4 of these were treatment-naïve with the latter on treatment with hydroxyurea (*n* = 3) or anagrelide (*n* = 1) at the time of referral. Informative cases were subsequently reassigned the diagnosis of either prefibrotic PMF (*n* = 8; 57%) or were felt to be morphologically consistent with true WHO-defined ET (*n* = 6; 43%); exposure to therapeutic agents at the time of bone marrow sampling was balanced between them (*n* = 2 each). Comparison of these two distinct histopathological patterns, i.e. true ET vs reassigned prefibrotic PMF, was respectively characterized by lower (median 35%, range 30–50) vs higher (median 65%, range 40–80) bone marrow cellularity (*P* < 0.001), ET (*n* = 6) vs PMF (*n* = 8) consistent megakaryocyte morphology (*P* < 0.001) and presence of trilineage proliferation (0% vs 100%; *P* < 0.001); in contrast, the degree of reticulin fibrosis was similar between the two (*P* = 0.1) (Supplemental Table 1). The reassigned prefibrotic PMF (*n* = 8), vs confirmed ET (*n* = 6), cases presented platelet counts consistently <1000 × 10^9^/l (100% vs 67%; *P* = 0.06), a higher frequency of increased serum levels of lactate dehydrogenase (LDH) (60% vs 0%; *P* = 0.02), higher likelihood of having hemoglobin levels below the sex-adjusted reference range values (29% vs 0%; *P* = 0.1), leukoerythroblastosis (14% vs 0%; *P* = 0.2), constitutional symptoms (13% vs 0%; *P* = 0.2), and a higher incidence of thrombosis history at presentation (38% vs 0%; *P* = 0.04) (Table 1). Interestingly, reassigned prefibrotic PMF also displayed a narrower *MPL* mutational spectrum compared to those confirmed as ET (*MPL*W515L/K incidence 100% vs 60%; *P* = 0.04). The incidences of abnormal karyotype and high molecular risk mutations (*ASXL1*, *SRSF2*, and *U2AF1*) were similarly low between the two groups (Table 1). Over a median follow-up of 8 (reassigned prefibrotic PMF) and 10 years (true *MPL*-mutated ET), we documented a higher incidence of thrombosis after diagnosis in prefibrotic PMF (25% vs 0%; *P* = 0.1) but similar rates of leukemic transformation (0% for both) and fibrotic progression (38% vs 33%; *P* = 0.87).

**Table 1:** Comparison of presenting features and outcomes in three *MPL*-mutated cohorts: *MPL*-mutated essential thrombocythemia having undergone central pathology review and either confirmed as essential thrombocythemia (*n* = 6) or re-classified as primary myelofibrosis (*n* = 8) and *MPL*-mutated primary myelofibrosis (*n* = 54)

Variables	*MPL*-mutated ET confirmed by central review (*n* = 6)	*MPL*-mutated “ET” re-classified as PMF by central review (*n* = 8)	*MPL*-mutated PMF (*n* = 54)	*P*-value All groups	*P*-value ET confirmed vs re-classified as PMF
Age at diagnosis, years; median (range)	69 (57–87)	69 (56–77)	65 (29–86)	0.57	0.89
Gender, male; *n* (%)	3 (50)	5 (63)	29 (54)	0.87	0.64
Age > 70 years; *n* (%)	1 (17)	4 (50)	17 (31)	0.40	0.19
Leukocytes, ×10^9^/l; median (range) “*N*” evaluable = 66 (97%)	6.4 (4.1–10.6)	7.3 (6–10.1)	6.2 (1.7–52.9)	0.79	0.42
Leukocytes ≥ 11 × 10^9^/l; *n* (%)	0 (0)	0 (0)	14 (26)	**0.04**	N/C
Hemoglobin, g/dl; median (range) “*N*” evaluable = 66 (97%)	14.1 (12.1–14.8)	13.5 (12.6–14.5)	9.9 (6.4–13.2)	**<** **0.0001**	0.37
Mild anemia, sex-adjusted^a^; *n* (%)	0 (0)	2 (29)	24 (44)	0.07	0.12
Moderate-severe anemia, sex-adjusted^a^; *n* (%)	0 (0)	0 (0)	29 (54)	**0.0003**	N/C
Platelets, ×0^9^/l; median (range) “*N*” evaluable = 67 (99%)	949 (800–1539)	850 (551–961)	183 (14–1371)	**<** **0.0001**	0.25
Platelets>1000 × 10^9^/l; *n* (%)	2 (33)	0 (0)	3 (6)	0.09	0.06
LDH (U/L); median (range) “*N*” evaluable = 58 (85%)	204 (130–226)	248 (157–452)	552 (195–1426)	**<** **0.0001**	0.25
LDH elevated above reference range; *n* (%)	0 (0)	3 (60)	47 (98)	**<** **0.0001**	0.02
RDW (%); median (range) “*N*” evaluable = 66 (97%)	14.4 (13.5–20.4)	14.5 (13.5–17.7)	19.3 (12.7–30.7)	**0.0002**	0.83
RDW above reference range; *n* (%)	2 (33)	2 (29)	49 (92)	**<** **0.0001**	0.85
Anisopoikilocytosis; *n* (%) “*N*” evaluable = 62 (91%)					
No	3 (50)	5 (71)	1 (2)	**<** **0.0001**	0.40
Slight	2 (33)	2 (29)	13 (27)		
Moderate	1 (17)	0 (0)	24 (49)		
Marked	0 (0)	0 (0)	11 (22)		
Dacryocytes; *n* (%) “*N*” evaluable = 57 (84%)					
No	5 (83)	7 (100%)	1 (2)	**<** **0.0001**	0.19
Slight	1 (17)	0 (0)	18 (41)		
Moderate	0 (0)	0 (0)	20 (46)		
Marked	0 (0)	0 (0)	5 (11)		
Leukoerythroblastic picture; *n* (%) “*N*” evaluable = 52 (76%)	0 (0)	1 (14)	34 (87)	**<** **0.0001**	0.25
Karyotype; *n* (%) “*N*” evaluable = 66 (97%)					
Normal	5 (83)	8 (100)	36 (69)	0.06	0.18
Abnormal	1 (17)	0 (0)	16 (31)		
Presence of very high risk karyotype^b^ “*N*” evaluable = 66 (97%)	0 (0)	0 (0)	1 (2)	0.79	N/C
Bone marrow reticulin fibrosis grade (initial report); median (range) “*N*” evaluable = 65 (96%)	0 (0–2)	0 (0–1)	3 (1–3)	**<** **0.0001**	0.85
Bone marrow reticulin fibrosis ≥grade 2; *n* (%)	0 (0)	1 (17)	33 (83)	**0.0001**	0.35
Central pathology review; “*N*” evaluated = 14					
Reticulin fibrosis (0–3+); median (range)	0 (0)	0 (0–3)	N/C	N/C	0.1
Bone marrow cellularity %; median (range)	35 (30-50)	65 (40–80)			**0.0005**
Megakaryocyte morphology ET vs PMF; *n* (%)	ET = 6 (100)	PMF = 8 (100)			**<0.0001**
Trilineage proliferation; yes or no	No = 6 (100)	Yes = 8 (100)			**<0.0001**
Constitutional symptoms present; *n* (%) “*N*” evaluable = 68 (100%)	0 (0)	1 (13)	12 (22)	0.21	0.27
Palpable splenomegaly; *n* (%) “*N*” evaluable = 62 (91%)	1 (17)	0 (0)	30 (63)	**0.0002**	0.18
History of thrombosis at or prior to diagnosis; *n* (%)	0 (0)	3 (38)	3 (6)	**0.03**	**0.04**
History of thrombosis after diagnosis; *n* (%)	0 (0)	2 (25)	5 (9)	0.25	0.11
Conventional risk stratification; “*N*” evaluable = 67 (99%)					
Low; *n* (%)	1 (17)	0 (0)	9 (17)	**<** **0.0001**	0.06
Intermediate; *n* (%)	5 (83)	4 (57)	N/A		
Intermediate-1; *n* (%)	N/A	N/A	18 (33)		
Intermediate-2; *n* (%)	N/A	N/A	24 (44)		
High; *n* (%)	0 (0)	3 (43)	3 (6)		
*MPL* mutation type“*N*” evaluable = 62 (91%)					
W515L/K	3 (60)	8 (100)	40 (82)	0.10	**0.04**
W515R	2 (40)	0 (0)	3 (6)	0.06	**0.04**
W515S	0 (0)	0 (0)	1 (2)	0.79	N/A
S505N	0 (0)	0 (0)	3 (6)	0.48	N/A
Other^c^	0 (0)	0 (0)	4 (8)	0.38	N/A
Number *MPL* mutations; median (range)	1 (1–3)	1 (1–1)	1 (1–2)	0.31	0.21
*MPL* allele burden; *n* (%) “*N*” evaluable = 10 (15%)					
<40%	N/A	N/A	3 (30)	N/C	N/C
40–80%			4 (40)		
>80%			3 (30)		
*ASXL1* mutation; *n* (%) “*N*” evaluable = 51 (75%)	0 (0)	0 (0)	15 (36)	**0.03**	N/C
*SRSF2* mutation; *n* (%)“*N*” evaluable = 50 (74%)	0 (0)	0 (0)	10 (24)	0.11	N/C
*U2AF1* mutation; *n* (%) “*N*” evaluable = 51 (75%)	0 (0)	0 (0)	6 (14)	0.29	N/C
*IDH1* mutation; *n* (%) “*N*” evaluable = 42 (62%)	0 (0)	0 (0)	1 (3)	0.78	N/C
*IDH2* mutation; *n* (%) “*N*” evaluable = 42 (62%)	1 (25)	0 (0)	3 (9)	0.40	0.18
*EZH2* mutation; *n* (%) “*N*” evaluable = 42 (62%)	0 (0)	0 (0)	2 (6)	0.61	N/C
*TET2* mutation; *n* (%) “*N*” evaluable = 19 (28%)	1 (25)	1 (20)	2 (20)	0.98	0.86
*SF3B1* mutation; *n* (%) “*N*” evaluable = 21 (31%)	0 (0)	1 (20)	1 (8)	0.52	0.26
*RUNX1* mutation; *n* (%) “*N*” evaluable = 19 (28%)	0 (0)	0 (0)	0 (0)	N/C	N/C
Fibrotic progression; *n* (%)	2 (33)	3 (38)	N/A	N/A	0.87
Leukemic progression; *n* (%)	0 (0)	0 (0)	6 (11)	0.23	N/C
Follow-up in years;median (range)	10 (5–15)	8 (1-18)	3 (0.05-23)	**0.005**	0.79
Deaths; *n* (%)	2 (33)	4 (50)	35 (65)	0.27	0.53

*ET* essential thrombocythemia, *PMF* primary myelofibrosis, *MPL* myeloproliferative leukemia virus oncogene, *LDH* lactate dehydrogenase, *RDW* red cell distribution width, *N/A* not available, *N/C* not computable

^a^Mild and moderate-severe anemia, sex-adjusted were defined respectively as hemoglobin values ≥10 g/dl but below sex-adjusted lower limit of normal (13.5 in men and 12.0 in women in our center) and <10 g/dl in women and <11 g/dl in men

^b^ Very high risk karyotype includes single or multiple abnormalities of −7, i(17q), inv(3)/3q21, 12p-/12p11.2, 11q−/11q23, or other autosomal trisomies not including +8/+9 (e.g., +21, +19)

^c^Other *MPL* mutations identified included: S204P, P440L, p.Tryp515*, unspecified insertion/deletion at amino acid position 515, and p.Leu498_His499insValIleAlaLeu

Bold values represent *p*-values that are statistically significant

When all 665 ET patients were assessed for myelofibrosis-free survival, *MPL*-mutated cases (prior to central review) displayed significantly worse outcome compared to patients with other driver mutations with a myelofibrosis transformation rate of 33% compared to 11%, 15%, and 21% in triple negative, *JAK2*, and *CALR*-mutated cohorts, respectively (*P* = 0.04; Fig. 1a). Median overall survival rates in confirmed *MPL*-mutated ET, ET re-classified as PMF, and *MPL*-mutated PMF were not reached, 11.6 and 5.3 years, respectively (confirmed ET vs PMF, *P* = 0.01; ET re-classified vs PMF, *P* = 0.04; confirmed ET vs ET re-classified as PMF, *P* = 0.54) (Fig. 1b).

**Fig. 1:**
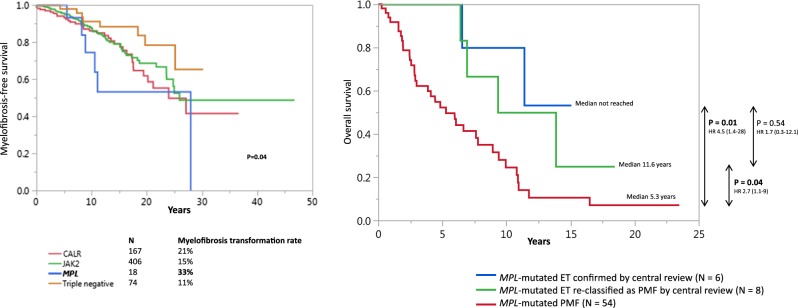
**1a** Myelofibrosis-free survival data in 665 patients with essential thrombocythemia stratified by driver mutational status. **1b** Survival data in 14 centrally re-reviewed patients with *MPL*-mutated essential thrombocythemia; morphologically confirmed vs re-classified as primary myelofibrosis vs *MPL*-mutated primary myelofibrosis

The current study suggests that the majority of routinely assigned cases of *MPL*-mutated ET probably represent prefibrotic PMF when morphologically scrutinized. We fully acknowledge the limitations inherent to this report including its retrospective nature and the limited number of informative cases, and our data require further validation. These conditions notwithstanding, we have documented, even prior to central pathology review, a significantly higher rate of fibrotic progression in *MPL*-mutated ET compared to patients with other driver mutations. While not all reports are consistent in this regard^14^, our data remain aligned with the majority of large scale, mature studies on the subject^8,9^. After central pathology review, the similar rates of fibrotic progression between morphologically confirmed *MPL*-mutated ET and those reassigned as prefibrotic PMF further suggest the latter to be biologically more akin to PMF. Correspondingly, when clinical correlates were considered in concert with morphologic assessment, patients re-classified as *MPL*-mutated prefibrotic PMF presented more frequent features consistent with their morphological re-allocation including presence of hemoglobin below sex-adjusted norms and LDH concentrations above reference range, all of which were consistently and conspicuously absent in those conserving their true *MPL*-mutated ET designation. Although rates of leukemic transformation and overall survival estimates did not differ substantially between the two groups, previous data have disclosed a markedly relevant influence of accurate morphological diagnosis on survival in ET^10^ and we believe this distinction remains an important one. Consequently, the exceeding infrequency of true *MPL*-mutated ET should at the very least confront clinicians with the possibility that some, if not most, of these cases correspond to prefibrotic PMF and prompt closer consideration and diagnostic revision when warranted.

## Electronic supplementary material

Supplemental Table 1
