# Supplementary material for: MPL-mutated essential thrombocythemia: a morphologic reappraisal
Source: Blood Cancer J. 2018 Nov 20;8(12):121. doi: 10.1038/s41408-018-0159-3 (PMC6246562; doi:10.1038/s41408-018-0159-3)
Supplement: Supplementary file 1 — Supplemental Table 1 [file 41408_2018_159_MOESM1_ESM.docx]

| **Variables**  **Patients** | **Age at diagnosis, years** | **Gender** | **Leukocytes***  **(x 10^9^/L)** | **Hemoglobin* (g/dL)** | **Platelets***  **(x 10^9^/L)** | **LDH* (U/L)**  **Reference range**  **112-257 U/L** | **RDW***  **(%)**  **Reference range**  **11.8-15.5%** | **Peripheral smear:**  **Anisopoikilocytosis Dacryocytes Leukoerythroblastic** | | | **Karyotype** | ***MPL* mutation type(s)** | **Additional mutations** | **IPSET risk category** |
| --- | --- | --- | --- | --- | --- | --- | --- | --- | --- | --- | --- | --- | --- | --- |
| Patient 1 | 56 | Female | N/A | N/A | N/A | N/A | N/A | N/A | N/A | N/A | 46,XX[20] | W515K | N/A | N/A |
| Patient 2 | 63 | Male | N/A | 12.6 | 850 | 452 | 17.7 | No | No | No | 46,XY[20] | W515L | N/A | High |
| Patient 3 | 71 | Male | 6.6 | 13.5 | 930 | 248 | 13.6 | No | No | No | 46,XY[20] | W515L | No | Intermediate |
| Patient 4 | 66 | Female | 9 | 12.6 | 834 | 157 | 16.3 | Slight | No | No | 46,XX[20] | W515L | No | High |
| Patient 5 | 77 | Female | 7.4 | 13.7 | 930 | N/A | 14.5 | No | No | Yes | 46,XX[20] | W515L | No | Intermediate |
| Patient 6 | 59 | Male | 7.2 | 13.3 | 777 | 184 | 13.5 | Slight | No | No | 46,XY[20] | W515L | *TET2* | Intermediate |
| Patient 7 | 76 | Male | 10.1 | 14.3 | 961 | 317 | 15.4 | No | No | No | 46,XY,t(6;11)(p21.3;q23)[1]/46,XY[19] | W515L | N/A | Intermediate |
| Patient 8 | 74 | Male | 6 | 14.5 | 551 | N/A | 13.6 | No | No | No | 46,XY[20] | W515K | *SF3B1* | High |
| Patient 9 | 69 | Male | 10.6 | 14.4 | 1124 | 130 | 14.3 | No | No | No | 46,XY[N/A] | W515R | N/A | Intermediate |
| Patient 10 | 69 | Male | 6.4 | 14.8 | 993 | N/A | 14.1 | Slight | No | No | 46,XY[20] | W515L | No | Intermediate |
| Patient 11 | 57 | Female | 4.1 | 9.1 | 1539 | 226 | 20.4 | Moderate | Occasional | No | 46,XX,t(1;21)[20] | W515L | No | Low |
| Patient 12 | 87 | Male | 9.5 | 14.1 | 803 | 213 | 14.4 | No | No | No | 46,XY[20] | W515L | *TET2* | Intermediate |
| Patient 13 | 61 | Female | 5.4 | 12.1 | 800 | 177 | 17.4 | Slight | No | No | 46,XX[20] | N/A | *IDH2* | Intermediate |
| Patient 14 | 69 | Female | 6.3 | 13.9 | 904 | 204 | 13.5 | No | No | No | 46,XX[20] | W515R plus 2 stop codons (p.Trp515*) | N/A | Intermediate |

**Supplemental Table 1. Detailed laboratory, morphological, and clinical characteristics and outcomes in 14 centrally re-reviewed cases of *MPL*-mutated essential thrombocythemia**

| **Variables**  **Patients** | **Central BM**  **pathology review** | **Reticulin fibrosis**  **(0 to 3+)** | **Bone marrow cellularity (%)** | **Mega-karyocyte**  **morphology** | **Trilineage proliferation** | **Re-classification following central review** | **Palpable splenomegaly at diagnosis** | **Constitutional symptoms**  **at diagnosis** | **Thrombotic event at/prior to diagnosis** | **Thrombotic event post- diagnosis** | **Fibrotic**  **transformation** | **Leukemic**  **transformation** | **Median follow- up (years)** | **Status last follow-up** |
| --- | --- | --- | --- | --- | --- | --- | --- | --- | --- | --- | --- | --- | --- | --- |
| Patient 1 | Yes | 3 | 70 | PMF | Yes | PMF | No | Absent | No | No | Yes | No | 18.4 | Alive |
| Patient 2 | Yes | 0 | 70 | PMF | Yes | PMF | No | Present | Yes (arterial) | Yes (arterial) | Yes | No | 6.9 | Dead |
| Patient 3 | Yes | 0 | 60 | PMF | Yes | PMF | No | Absent | No | No | Not documented | No | 5.7 | Alive |
| Patient 4 | Yes | 1 | 80 | PMF | Yes | PMF | No | Absent | Yes (arterial) | No | Not documented | No | 11.6 | Alive |
| Patient 5 | Yes | 0 | 60 | PMF | Yes | PMF | No | Absent | No | No | Not documented | No | 13.8 | Dead |
| Patient 6 | Yes | 0 | 50 | PMF | Yes | PMF | No | Absent | No | Yes (arterial) | Yes | No | 9.3 | Dead |
| Patient 7 | Yes | 0 | 40 | PMF | Yes | PMF | No | Absent | No | No | Not documented | No | 1.3 | Alive |
| Patient 8 | Yes | 1 | 70 | PMF | Yes | PMF | No | Absent | Yes (arterial + venous) | No | Not documented | No | 6.4 | Dead |
| Patient 9 | Yes | 0 | 40 | ET | No | ET | No | Absent | No | No | Not documented | No | 12.3 | Alive |
| Patient 10 | Yes | 0 | 50 | ET | No | ET | No | Absent | No | No | Not documented | No | 5.1 | Alive |
| Patient 11 | Yes | 0 | 30 | ET | No | ET | Yes | Absent | No | No | Not documented | No | 11.4 | Dead |
| Patient 12 | Yes | 0 | 30 | ET | No | ET | No | Absent | No | No | Not documented | No | 6.5 | Dead |
| Patient 13 | Yes | 0 | 40 | ET | No | ET | No | Absent | No | No | Yes | No | 15 | Alive |
| Patient 14 | Yes | 0 | 30 | ET | No | ET | No | Absent | No | No | Yes | No | 8.3 | Alive |

Abbreviations: LDH, lactate dehydrogenase; RDW, red cell distribution width; *MPL*, Myeloproliferative leukemia virus oncogene; IPSET, International Prognostic Score in essential thrombocythemia; ET, essential thrombocythemia; PMF, primary myelofibrosis; N/A, not available; BM, bone marrow.

* All laboratory parameters were obtained at time of diagnosis.
